# Supplementary material for: Beyond Spectral Resolution in Nanophotonic Sensing: Picometer-Level Precision with Multispectral Readout
Source: ACS Nano. 2025 Jul 21;19(30):27515–25. doi: 10.1021/acsnano.5c06561 (PMC12333399; doi:10.1021/acsnano.5c06561)
Supplement: Supplementary file 1 [file nn5c06561_si_001.pdf]

Supplementary Information: Beyond spectral resolution in nanophotonic sensing: Picometer-level precision with multispectral readout

M.S. Cano-Velázquez,[*,†,‖] S. Buntinx,[†,‖] A.L. Hendriks,[†,‖] A. van Klinken,[†,‖] C. Li,[†,‖] B.J. Heijnen,[†] M. Dolci,[†] L. Picelli,[†,#] M.S. Abdelkhalik,[†] P. Sevo,[‡] M. Petruzzella,[†,‡] F. Pagliano,[†,‡,¶] K.D. Hakkel,[†,#] D.M.J. van Elst,[†] P.J. van Veldhoven,[†] E. Verhagen,[†,§] P. Zijlstra,[†] and A. Fiore[†]

†*Department of Applied Physics and Science Education, and Eindhoven Hendrik Casimir Institute, Eindhoven University of Technology, 5600 MB, Eindhoven, The Netherlands*
‡*MantiSpectra B.V.; Eindhoven, 5612 AE, The Netherlands*
¶*nanoPHAB B.V.; Eindhoven, 5612 AP, The Netherlands*
§*Center for Nanophotonics, AMOLF, Science Park 104, 1098 XG, Amsterdam, The Netherlands*
‖*These authors contributed equally to this work.*
⊥*Present address: College of Information Science and Electronic Engineering, Zhejiang University; Hangzhou, 310027, China and Research Center for Intelligent Optoelectronic Computing, Zhejiang Lab; Hangzhou, 311121, China*
#*Present address: MantiSpectra B.V.; Eindhoven, 5612 AE, The Netherlands*

E-mail: m.s.cano.velazquez@tue.nl

Sensitivity and Cramér-Rao lower bound

Derivation of the Cramér-Rao lower bound

Here we derive the Cramér-Rao lower bound (CRLB) for wavelength imprecision in the general case of N spectral measurements. For this estimation problem, the CRLB is given by:[1]

$$\sigma_{\lambda_s}^{CR} = \sqrt{\frac{1}{-E\left[\frac{\partial^2}{\partial\theta^2}\ln p_\theta(\overrightarrow{s};\theta)\right]}} \quad (1)$$

where $p_\theta(\overrightarrow{s};\theta)$ is the probability density function for the observable $\overrightarrow{s} = \begin{bmatrix} I_1 \\ \dots \\ I_N \end{bmatrix}$ (where I_i are the detector photocurrents), given the unknown parameter $\theta = \lambda_S$.

Assuming that the photocurrent of each detector follows a Gaussian distribution with average $\overline{I_i}$ and standard deviation σ_i, and that the photocurrents are uncorrelated, i.e. $p_{\lambda_S}(I_1, \dots I_N) = p_{\lambda_S}(I_1)\dots p_{\lambda_S}(I_N)$ and $p_{\lambda_S}(I_i) = \frac{1}{\sigma_i\sqrt{2\pi}} e^{-\frac{(I_i-\bar{I}_i)^2}{2\sigma_i^2}}$, we obtain: $\frac{\partial}{\partial\lambda_S}\ln p_{\lambda_S}(\overrightarrow{s};\lambda_S) = \frac{I_1-\bar{I}_1}{\sigma_1^2}\frac{\partial\bar{I}_1}{\partial\lambda_S} + \dots + \frac{I_N-\bar{I}_N}{\sigma_N^2}\frac{\partial\bar{I}_N}{\partial\lambda_S}$, $\frac{\partial^2}{\partial\lambda_S^2}\ln p_{\lambda_S}(\overrightarrow{s};\lambda_S) = -\frac{1}{\sigma_1^2}\left(\frac{\partial\bar{I}_1}{\partial\lambda_S}\right)^2 + \frac{I_1-\bar{I}_1}{\sigma_1^2}\frac{\partial^2\bar{I}_1}{\partial\lambda_S^2} + \dots - \frac{1}{\sigma_N^2}\left(\frac{\partial\bar{I}_N}{\partial\lambda_S}\right)^2 + \frac{I_N-\bar{I}_N}{\sigma_N^2}\frac{\partial^2\bar{I}_N}{\partial\lambda_S^2}$ and $\sigma_{\lambda_S}^{CR} = \frac{1}{\sqrt{\sum_{i=1}^{N}\frac{1}{\sigma_i^2}\left(\frac{\partial\bar{I}_i}{\partial\lambda_S}\right)^2}}$

In the case that the detector photocurrents have the same variance σ_I, we obtain Eq. 1 of

the main text:

$$\sigma_{\lambda_s}^{\mathrm{CR}} = \frac{\sigma_{\mathrm{I}}}{\sqrt{\sum_{i=1}^{\mathrm{N}} \left(\frac{\partial \bar{\mathrm{I}}_{\mathrm{i}}}{\partial \lambda_{\mathrm{S}}}\right)^2}} \quad (2)$$

Approximate expressions for the Cramér-Rao lower bound

The CRLB on the wavelength estimation (Eq. 1 in the primary manuscript) depends on the derivatives of the photocurrents: $\frac{\partial I_i}{\partial \lambda_S} = \int R_i(\lambda) \frac{\partial R_S}{\partial \lambda_S} P_\lambda(\lambda) d\lambda$. To provide simple estimates for these, we approximate the responsivity as $R_i(\lambda) \simeq \frac{e}{hc} \lambda_S A(\lambda)$, where $A(\lambda)$ is the absorptance of the detector (including the filtering structure), h the Planck's constant, and e is the electron charge. We assume that the power spectral density is constant, $P_\lambda(\lambda) = P_\lambda$. We also assume that $R_S(\lambda; \lambda_S)$ has a spectral feature (*e.g.* a peak or dip) centered at λ_S and that it translates along the wavelength axis according to λ_S. We can then write $\left|\frac{\partial I_i}{\partial \lambda_S}\right| = \frac{e}{hc} \lambda_S P_\lambda \left|\int A_i(\lambda) \frac{\partial R_S}{\partial \lambda_S} d\lambda\right| = \frac{e}{hc} \lambda_S P_\lambda \left|S_i(\lambda_S)\right|$, where $S_i(\lambda_S) \equiv \int A_i(\lambda) \frac{\partial R_S(\lambda;\lambda_S)}{\partial \lambda_S} d\lambda = -\int A_i(\lambda) \frac{\partial R_S(\lambda;\lambda_S)}{\partial \lambda} d\lambda$ is an adimensional sensitivity. In the ideal case of no optical loss, assuming that A_i and R_S consist of single Lorentzian lines centered at λ_i and λ_S, with the same full-width half-maximum $\Delta\lambda_{FWHM}$, it is easy to show that $S_i(\lambda_S)$ is zero for $\lambda_i = \lambda_S$ and takes a maximum value of $\sim$0.5 (independent of $\Delta\lambda_{FWHM}$) for $\lambda_i \simeq \lambda_S \pm \Delta\lambda_{FWHM}/2$, *i.e.* when the readout channels are positioned on either side of the sensor resonance. Reducing or increasing the width of the readout resonances reduces the peak value of S. This shows that the highest sensitivity and lowest wavelength imprecisions are obtained for matched sensor/readout linewidths. Generally, as A_i and R_S are bound between 0 and 1, each spectral band, where A_i and R_S both show a peak, can contribute at most 1 to the sensitivity, independent of their widths.

In the simplest case of single-channel readout, the Cramér-Rao bound is given by $\sigma_{\lambda_S}^{CR} = \frac{\sigma_I}{\left|\frac{\partial \bar{I}}{\partial \lambda_S}\right|} = \frac{\sigma_I}{\frac{e}{hc} \lambda_S P_\lambda |S_i(\lambda_S)|} = \frac{P_{min}}{P_\lambda |S_i(\lambda_S)|}$, where P_{min} is the detector's noise-equivalent power. For a single, optimally detuned readout channel, ($|S_i(\lambda_S)| \sim 0.5$) and $\sigma_{\lambda_S}^{CR} = 2P_{min}/P_\lambda$, which has a simple physical interpretation: The minimum wavelength shift $\delta\lambda_{min}$ that can be measured is such that the power of the source in that wavelength interval is of the order of the minimum power the detector can measure, $P_\lambda \delta\lambda_{min} \sim P_{min}$ (the additional factor 2 is due to the use of a single sideband). Any loss in the optical system and readout (including the loss due to the spatial multiplexing in the detector array and any detector inefficiency) can be incorporated into P_λ, which then becomes the power spectral density incident on the detector. By placing a second detector at the opposite side of the sensor resonance, the CRLB is slightly improved to $\sigma_{\lambda_S}^{CR} = \frac{\sigma_I}{\sqrt{2}\left|\frac{\partial I_i}{\partial \lambda_s}\right|}$. However, as seen from Eq. 1 of the primary manuscript, splitting up the readout in more channels with smaller linewidth (*i.e.* moving towards a spectrometer-based read-out) tends to degrade the wavelength imprecision, as the photocurrent scales with the linewidth. In practice, additional optical loss is usually associated with narrower linewidth (*e.g.* due to the need of reducing the étendue), which makes the scaling even worse.

Case of shot noise

The considerations of the primary manuscript apply to the case where thermal noise in the readout circuit is dominant, which is the most likely situation in a practical application setting. For dominating shot noise (in the limit of large photon numbers), Eq. 1 of the primary manuscript is modified to

$$\sigma_{\lambda_S}^{CR} = \frac{1}{\sqrt{\sum_{i=1}^{N} \frac{1}{\sigma_{I_i}^2} \left(\frac{\partial I_i}{\partial \lambda_S}\right)^2}} = \frac{\sqrt{2e\Delta f}}{\sqrt{\sum_{i=1}^{N} \frac{1}{I_i} \left(\frac{\partial I_i}{\partial \lambda_S}\right)^2}} \quad (3)$$

(where Δf is the measurement bandwidth), from which we see that, in an ideal optical system, the wavelength imprecision is independent

of the number of readout channels. However, in both dispersive spectrometers and multispectral arrays, improving the spectral resolution requires reducing the étendue, so that using more spectral channels with improved resolution leads to a degradation of the imprecision.

Generalization of the Cramér-Rao lower bound to more complex spectral changes

We have so far referred to the estimation of the wavelength λ_S, assuming that the parameter of interest x is mapped into a spectral shift of a single resonance, as in most existing resonant sensors. However, in a more general statement of the sensing problem, the lower bound on the imprecision in the parameter x is given (for thermal noise) by

$$\sigma_x^{CR} = \frac{\sigma_1}{\sqrt{\sum_{i=1}^{N} \left(\frac{\partial I_i}{\partial x}\right)^2}} \tag{4}$$

where $\frac{\partial I_i}{\partial x} = \int R_i(\lambda) \frac{\partial R_S}{\partial x} P_\lambda(\lambda) d\lambda$ can describe more general changes in the spectrum (*e.g.* increased/decreased reflection in certain bands). This allows benchmarking the performance of different sensing systems, including those where the transducer and the readout unit have more complex spectral lineshapes.

This approach based on the CRLB is very powerful as it allows comparing the ultimate performance of a variety of optical sensing systems, independently of the fitting/data analysis methods used to build prediction models.

Temperature sensing experiments

Temperature sensing time trace

In Fig. 1, the time trace of the wavelength, calculated using the OSA, and estimated with the multispectral readout, is presented. The temperature was systematically increased from 25 °C to 65 °C and then decreased from 60 °C to 30 °C, following the method described in the Methods section. The plot illustrates that the wavelength estimated by the multispectral readout closely matches the OSA measurements, not only during the plateaus but also during the transient periods. Furthermore, the inset demonstrates that the multispectral readout yields more accurate results.

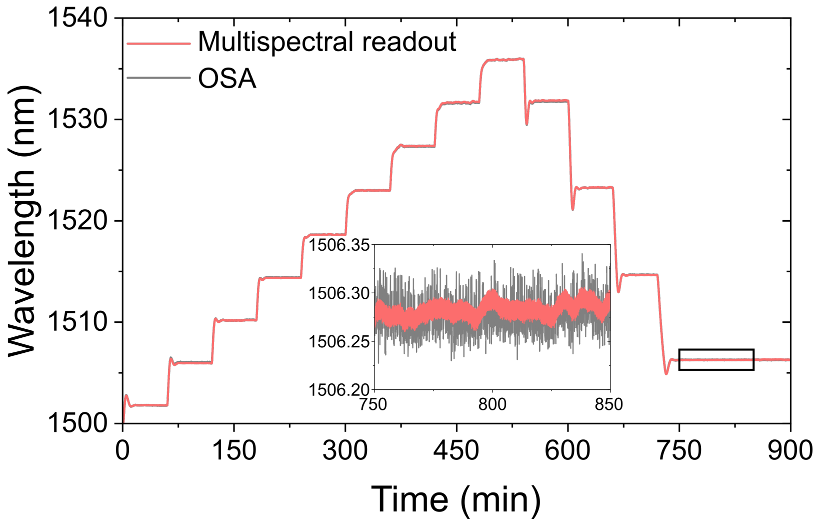

Figure 1: Time trace of the resonance wavelength obtained from the OSA and multispectral readout during a temperature experiment. The inset provides a zoomed-in view from 750 to 850 minutes to highlight the imprecision of both approaches.

Cramér-Rao lower bound in the experimental setting

The CRLB for wavelength estimation shown in Fig. 1(d) of the primary manuscript refers to the case of Lorentzian lineshapes for the read-out channels and an ideal Fabry-Perot cavity. To compare it to the experimental wavelength imprecision, we calculate the CRLB (Eq. 1 of the primary manuscript) with the sensitivities measured in the temperature sensing experiment (slopes of the counts vs wavelength data in Fig. 3(d) of the primary manuscript), and the experimental SNR corresponding to an integration time of 4.5 s. The resulting $\sigma_{\lambda_S}^{CR}$ (Fig. 2) is a function of wavelength, as the sensitivities vary with wavelength. At the temperature and wavelength (T=30 $°C$, λ=1506.3 nm), for which the Allan deviation of Fig. 3(d) was measured, we obtain $\sigma_{\lambda_S}^{CR}$=0.48 pm, which is very close to the experimental wavelength imprecision of 0.6 pm (minimum of Allan deviation of Fig. 3(d)). This proves that the estimation algorithm works optimally also with experimental data and provides a wavelength estimation with imprecision close to the fundamental CRLB limit.

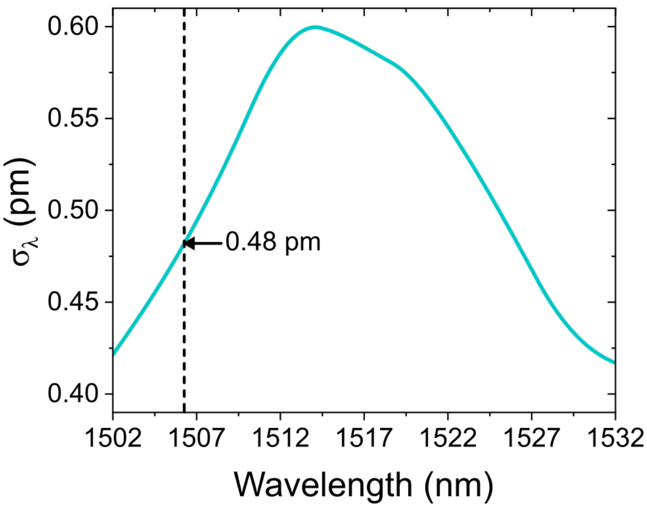

Figure 2: CRLB calculated from the sensitivities measured in the temperature sensing experiment and the experimental SNR corresponding to an integration time of 4.5 s.

Use of a light-emitting diode as a light source

To evaluate the feasibility of using a different light source for the proposed approach, a light-emitting diode (LED) was used instead of a halogen lamp in similar experiments to those described in the primary manuscript. Figure 3 illustrates the Allan deviation obtained when a Fabry-Pérot temperature sensor is used in combination with an LED (λ_C = 1550 nm, FHWM = 102 nm, P = 138 μW coupled to the fiber). The plot demonstrates that using the LED as the light source allows for a minimum wavelength imprecision of 1.4 pm and a minimum temperature imprecision of 1.7 mK. Although these values are slightly higher than those obtained with the halogen lamp, they are still comparable to the imprecision of the reference thermistor.

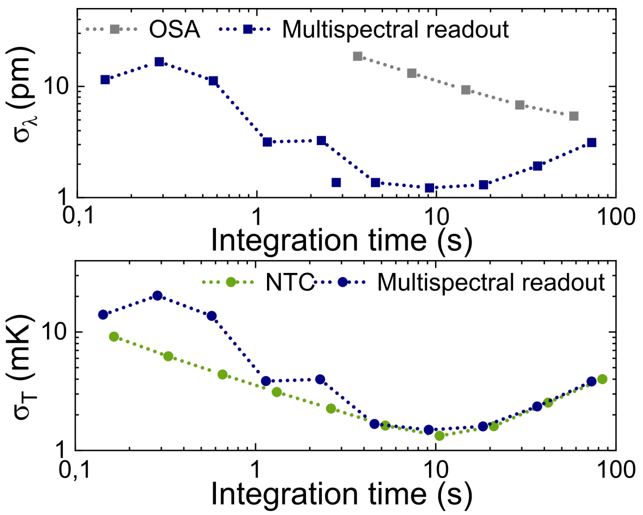

Figure 3: Allan deviation analysis of wavelength and temperature for multispectral readout, Optical Spectrum Analyzer (OSA), and reference temperature sensor (NTC) when a light-emitting diode (LED) is used as the light source.

Biosensing experiments

Control experiment

To prove the specificity of the functionalization of the biosensor, a control experiment was carried out on a nominally identical copy of the

PhC device. The results are depicted in Fig. 4. The surface was functionalized in the same way (see Results/Experimental Section of main text) with an IgG in PBS solution by physisorption (300 nM, 15 – 60 min) and a blocking step with a casein solution (75 – 95 minutes). After rinsing with PBS (until 100 min), the biosensor surface is again exposed to the same IgG in PBS solution (100 – 150 min). From the measured OSA spectrum, it is evident that there is no wavelength shift due to the exposure to biomolecules as to be expected for unspecific interactions. Consequently, the interaction with the target molecule anti-IgG in the next step of the assay (100 – 240 min) shows a clear response that corresponds to a specific interaction.

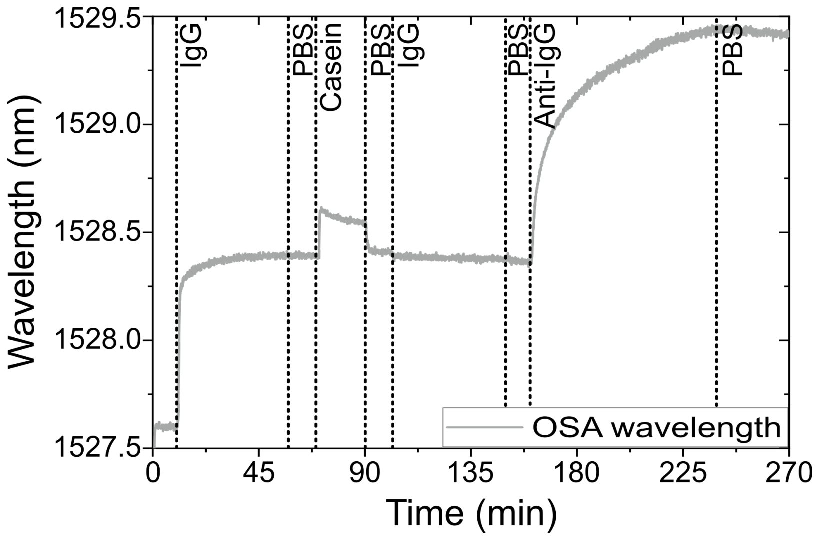

Figure 4: Time trace of the resonance wavelength obtained from the OSA during a biosensing experiment, where the functionalized sensor is exposed to IgG and subsequently to Anti-IgG to assess the specificity of the functionalization.

References

(1) Kay, S. M. *Fundamentals of statistical signal processing: Estimation theory v.1*; Prentice Hall international editions; Prentice-Hall: London, England, 1993.
